# Clinical study of low-frequency acupoint electrical stimulation to improve thumb-to-finger movements after stroke: A randomized controlled trial

**DOI:** 10.1097/MD.0000000000035755

**Published:** 2023-11-24

**Authors:** Xue Xia, Xu Dong, Hong Huo, Ying Zhang, Jing Song, Dongyan Wang

**Affiliations:** a Heilongjiang University of Chinese Medicine, Harbin, China; b Second Affiliated Hospital of Heilongjiang University of Chinese medicine, Harbin, China.

**Keywords:** low-frequency acupoint electrical stimulation, muscle function, stroke, surface electromyography, thumb-to-finger movement

## Abstract

**Objective:**

To examine the effect of low-frequency acupoint electrical stimulation (LFES) on the surface electromyographic (sEMG) signals of the thumb-to-finger movement muscles in stroke patients, and to evaluate the clinical efficacy of LFES on hand function recovery after stroke.

**Methods:**

Sixty patients who met the inclusion criteria were randomly assigned to a LFES group or an electroacupuncture (EA) group, with 30 patients in each group. Both groups received conventional treatment, and the EA group was treated with acupoints from the book of Acupuncture and Moxibustion, while the LFES group was treated with acupoints from a previous study. The sEMG characteristic values (maximum value and RMS), Chinese Stroke Clinical Neurological Deficit Scale (CSS), Brunnstrom Motor Function Evaluation, Modified Ashworth Scale (MAS), Lindmark Hand Function Score and Lovett Muscle Strength Classification were measured before and after treatment.

**Results:**

After treatment, both groups showed improvement in sEMG characteristic values, Brunnstrom motor function score, Lindmark hand function score, and Lovett muscle strength classification compared with before treatment, and the improvement in the LFES group was significantly better than that in the EA group (*P *< .05). The CSS score and MAS classification of both groups decreased compared with before treatment, and the decrease in the LFES group was significantly better than that in the EA group (*P* < .05). The total effective rate of the LFES group was 92.86%, and that of the EA group was 79.31%. The difference between the 2 groups was statistically significant (*P* < .05).

**Conclusion:**

Both LFES and EA were effective in restoring thumb-to-finger movement function after stroke, as evidenced by the increased maximum value and root mean square values of the first dorsal interosseous muscle and the extensor pollicis brevis muscle, the decreased CSS score, the increased Brunnstrom motor function score, the decreased MAS classification, the increased Lindmark hand function score, and the increased Lovett muscle strength classification. However, LFES showed more obvious improvement and better efficacy than EA, which is worthy of clinical promotion.

## 1. Introduction

Stroke is a lesion caused by blockage or rupture of a blood vessel in the brain, resulting in ischemic and hypoxic necrosis of brain tissue, corresponding neurological deficits and consequent symptoms in the dominant area.^[1]^ In recent years, the incidence of stroke has been on the rise year by year,^[2]^ with episodes of dizziness and coma in mild cases and shock and paralysis in severe cases, and is of great concern because of its high incidence, disability and paralysis rates.^[3]^ Stroke can seriously affect the motor^[4]^ and sensory functions of the limbs,^[5]^ resulting in varying degrees of functional impairment, and the difficulty of recovery and poor healing can seriously affect patients’ daily life,^[6]^ making them physically and mentally exhausted.^[7]^ The recovery and reconstruction of upper limb function has always been the focus and difficulty of poststroke limb rehabilitation, and there are very few studies on the recovery of hand function. Most upper extremity motor functions depend on the hand, therefore, the recovery of upper extremity motor functions in poststroke patients will depend on the recovery of hand functions.

Thumb-to-finger is one of the basic movements that the hand can complete, and clinically rehabilitate through the opponent’s thumb-to-finger movement to restore certain hand functions. The thumb-to-finger process is actually the pinching process, during which the muscles involved are mainly the first interosseous dorsal muscle and the short thumb extensors, which move in concert to complete the above action. FLES is the application of low-frequency electrical currents to traditional acupuncture points, both to improve the internal state of nerve cells through electrical currents and to regulate the balance of yin and yang, unblock meridians, and promote the flow of qi and blood through acupoint stimulation.^[8]^ A program is set up to imitate or trigger normal muscle activity in order to restore or enhance the functional level of the stimulated muscle or muscle group. Flexor-extensor alternating FLES can effectively improve the abnormal flexor pattern, but also complement the treatment of simple stimulation of the extensor muscle, which is more in line with the science and integrity of the movement. By continuously improving the stimulation method and exploring different locations, it can cause different movements of the finger, making the treatment more targeted. In a previous study, the team found that FLES was effective in improving upper limb function,^[9]^ dorsal wrist extension,^[10]^ hand grasp,^[11]^ lateral hand pinch-related muscle^[12]^ function, and thumb motor function after stroke. Therefore, this study will take FLES to improve the precision of thumb-to-finger movements after stroke and compare it with traditional electroacupuncture (EA) therapy to investigate its clinical efficacy on improving the precision of thumb-to-finger movements after stroke, aiming to provide a new therapeutic idea for upper limb hand function rehabilitation.

## 2. Research methodology

### 2.1. Study design, clinical data, and sample size

**Figure 1.**
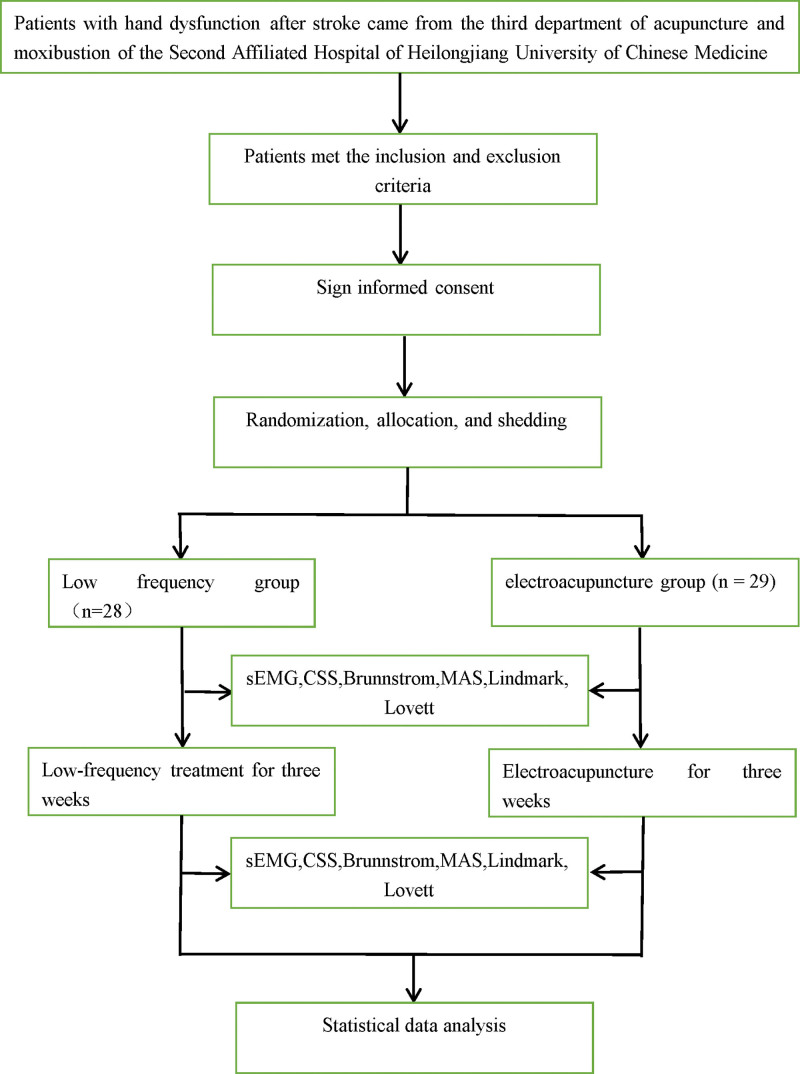
Flow chart of the study.

This randomized controlled trial selected patients with impaired hand function after stroke who were treated at the Acupuncture III Department of the Second Hospital of Heilongjiang University of Traditional Chinese Medicine from January 2022 to December 2022. This study was reviewed and approved by the Clinical Ethics Committee of the Second Hospital of Heilongjiang University of Chinese Medicine (approval number: [2022-K83]). The research process can be seen in Figure 1. The clinical trial registration number for this study is NCT06061731.

*Inclusion criteria*: ① Compliance with the diagnostic criteria of TCM and Western medicine. Diagnostic criteria of TCM: the diagnostic criteria of stroke disease were selected according to the Diagnostic Efficacy Criteria of TCM Diseases of the State Administration of Traditional Chinese Medicine.^[13]^ Western medical diagnostic criteria: refer to “Diagnostic points of various major cerebrovascular diseases in China 2019” stroke, and all patients were confirmed by head-related imaging.^[14]^ ② Age 35 to 75 years and duration of illness is 2 weeks to 3 months. ③ Impaired hand function with Lovett classification ≥ grade 2 and Modified Ashworth Scale (MAS) ≤ grade 2. ④ No organ dysfunction such as heart, liver, lung, kidney or blood circulation dysfunction. ⑤ Clear consciousness, no major impairment in intelligence, hearing or speech, and stable condition. ⑥ Patients voluntarily participated in this subject trial and signed the informed consent form.

*Exclusion criteria*: ① Presence of neurological or musculoskeletal disorders affecting functional recovery prior to the onset of the disease. ② Brainstem infarction, bilateral cerebral infarction or transient ischemic attack.

*Rejection criteria*: ① Cases in which treatment was not followed and clinical data information was incomplete, causing them to affect the evaluation and judgment of treatment effectiveness and safety. ② Persons who have had serious complications or whose condition has deteriorated rapidly during this treatment. ③ Have other conditions that are no longer suitable for treatment.

*Shedding criteria*: ① The information is missing and cannot be perfected, and it is difficult to determine the efficacy of the treatment. ② Those who have low compliance, do not cooperate with treatment or withdraw from the study on their own.

Sample size estimation, randomization method and allocation concealment, blind setting and implementation: In this study, 60 patients were included by referring to the relevant research literature,^[15]^ and 60 opaque envelopes numbered from 1 to 60 were prepared using a randomized grouping method, with a numbered slip of paper placed in each envelope. An envelope was randomly selected when patients were enrolled in the group, and according to the 30 random numbers generated by SPSS26.0 software, patients with corresponding numbers were divided into the low-frequency group and the rest into the EA group, and the 2 groups received treatment in a 1:1 ratio. Among them, 2 patients in the low-frequency group could not adhere to the treatment due to poor compliance, which affected the determination of efficacy and withdrew; 1 patient in the electro-acupuncture group withdrew from the treatment due to personal reasons, and finally 28 patients in the low-frequency group and 29 patients in the electro-acupuncture group, with 57 patients’ data counted to the final result. Blinding was implemented during the study, with subjects, evaluators and statistical analysts blinded to the details of the trial design so that they did not know which group was the low-frequency group and which group was the EA group. Only the applicator knows the difference between the groups, and neither the evaluator, nor the statistician is involved in the treatment process of the subjects.

### 2.2. Treatment protocol

Patients in both groups received conventional treatment in the acupuncture department, and the Chinese guidelines for primary prevention of cerebrovascular disease in the 2021 edition of the Chinese Multidisciplinary Expert Consensus on Stroke Disease Monitoring^[16]^ were referred to for interventions to prevent and control risk factors for cerebrovascular disease. Conventional treatment is upper limb rehabilitation training is to select appropriate exercise therapy and occupational therapy according to the degree of upper limb dysfunction and recovery stage of the patient, so as to improve the coordination, flexibility, speed and fine motor ability of the upper limb. The specific training methods are as follows: exercise therapy: including joint passive movement, joint rotation movement, upper limb suspension and placement, upper limb wall touching exercise, upper limb shooting ball, upper limb throwing wooden block, etc; Occupational therapy: including daily living ability training, finger separation and training, grasping and placing small balls, writing, playing basketball, clapping, etc.

EA group treatment: refer to the 13th Five-Year Plan textbook “Acupuncture and Moxibustion”^[17]^ for the selection of acupoints: Shuigou (DU26), Neiguan (PC6), Chize (LU5), Jiquan (HT1), Shousanli (LI10), and Hegu (LI4). Operation: the patient is placed in the supine position, the skin of the operated area is routinely disinfected with 75% alcohol, and the Huatuo brand sterile single-use acupuncture needle with the specification of 0.30 × 0.40mm is used for acupuncture. DU26 (shuigou) upward obliquely needling (insertion) 0.5 cun, perform reducing method, PC6 (Neiguan) straight needling (insertion) 1 cun, perform lifting and inserting reducing method, LU5 (Chize) straight needling (insertion) 1 cun, perform lifting and inserting reducing method to make the limb twitch, HT1 (Jiquan) stab avoiding the axillary artery straight needling (insertion) 0.5 cun, perform lifting and inserting reducing method to the extent that the patient’s upper limbs have a feeling of numbness and swelling and twitching. LI10 (Shousanli) and LI4 (Hegu) straight needling (insertion) 1 cun, perform increasing and reducing method. After the above acupuncture to deqi, choose KWD-808 Ⅰ type Indy brand pulse acupuncture treatment instrument, the waveform is continuous wave, the stimulation frequency is 2 Hz, the intensity to be tolerated by the patient. There should be muscle contraction at the acupuncture site during EA treatment. The treatment time should be 30 minutes each time, once a day, 6 days a week with 1 day off, for a total of 3 weeks.

**Figure 2.**
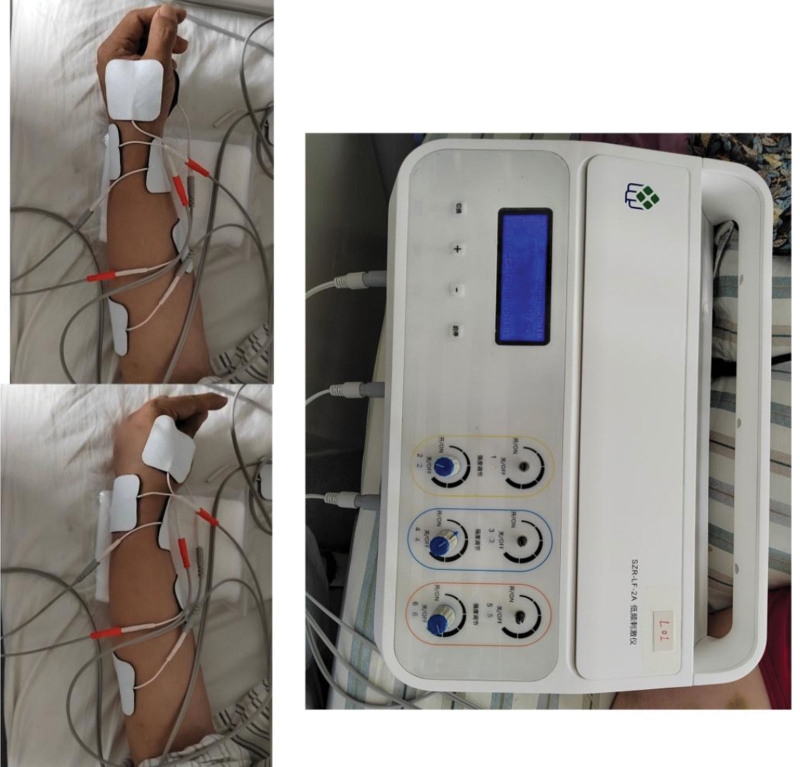
Treatment chart of low frequency group.

Low frequency group treatment: selected points: Group 1: Shousanli (LI10), Waiguan (SJ5); Group 2: Neiguan (PC6), Ximen (PC4); Group 3: Yuji (LU10), Hegu (LI4). Operation: The patient is seated with the shoulder joint slightly abducted, the elbow joint flexed, the forearm standing sideways on the table, and the hand slightly clenched in a fist. SZR-LF-2A low frequency stimulator and nonwoven electrode patch with the specification of 4 * 4 cm were selected. Routine sterilization of the application site before operation, low-frequency stimulation instrument and electrode patch connected, each group of wires connected to 2 patches, a total of 3 groups, respectively, placed on the selected acupuncture point, the first group of Shousanli, Waiguan, the second group of Neiguan, Ximen, the third group of Yuji, Hegu. The treatment frequency is selected as 50Hz, pulse width 0.3ms, waveform is intermittent waveform, stimulation intensity is tolerated by the patient, the instrument program is set to stimulate the first group of acupoints → second group → first group → third group, forming a set of programmed movements, cyclic operation, appearing alternate movements of flexor and extensor muscles, namely: wrist dorsal extension, five-finger extension → five-finger flexion → wrist dorsal extension, five-finger extension → thumb-index finger pair pinching, simulating fine movements Grasp of the hand, thumb-index finger pair pinch. The treatment course was the same as that of the EA group. The low-frequency treatment process can be seen in Figure 2.

### 2.3. Observed indicators

The following subjective and objective indicators were measured before and after treatment in both groups.

#### 2.3.1. Objective index measurement.

**Figure 3.**
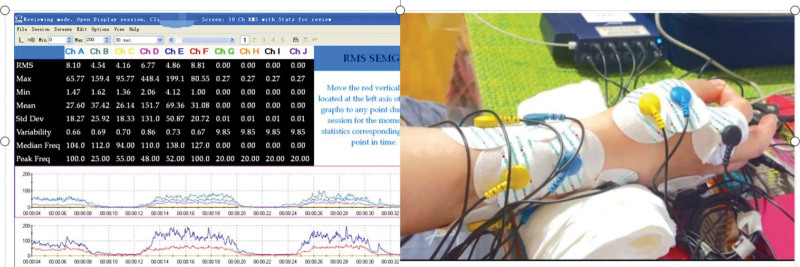
Surface EMG collection.

Surface electromyographic eigenvalues: 2 main aspects, maximum value (MAX), and root mean square value (RMS). MAX reflects the maximum contraction strength of the measured muscle. RMS is the root mean square of the EMG transient amplitude over time, which reflects the average change in EMG signal and is therefore used as an evaluation of muscle contraction performance. Surface EMG acquisition is shown in Figure 3.

#### 2.3.2. Subjective scale collection.

① *Chinese Stroke Clinical Neurological Deficit Scale (CSS) score*: In this study, the upper extremity and hand muscle strength and motor function were assessed to evaluate the efficacy of patients, and the consciousness, upper extremity and hand evaluation components were selected for assessment.

② *Brunnstrom method of motor function evaluation*: Grade I: no movement; Grade II: only weak flexion and extension; Grade III: hook grasp, but not finger extension; Grade IV: able to pinch laterally and thumb release, fingers can be extended semi-randomly and to a small extent; Grade V: can ball and column grasp, fingers extend simultaneously, but not individually; Grade VI: all grasps can be completed, but the speed and accuracy are worse than the noninvolved side.

③ *MAS*: Grade 0: no increase in muscle tone; Grade 1: mildly increased muscle tone, with minor resistance felt during passive flexion/extension to the maximum extent in grasping movements; Grade 1+: slightly increased muscle tone, with minor resistance felt during flexion/extension to more than 1/2 range in grasping movements; Grade 2: heavy resistance felt in most ranges of motion, but passive activities can be performed Grade 3: muscle tone is significantly elevated and passive activities are not easily performed; Grade 4: the affected portion of the limb exhibits tonic extension or flexion.

④ Lindmark hand function score: 0 points: gripping action cannot be completed; 1 point: gripping action can be completed, but cannot resist tiny resistance; 2 points: can hold an object for 5s, but cannot resist medium resistance, or grip is not standard, uncoordinated; 3 points: grip is normal, can hold an object against larger resistance for 5 seconds, and can release the hand like normal people, the total score is 24 points, the higher the score, the The higher the score, the better the hand function.

⑤ Lovett muscle strength classification: Grade 0: complete muscle paralysis, palpation muscle completely no contraction; Grade I: slight muscle contraction, but cannot cause joint movement; Grade II: can drive the joint horizontal activity, but cannot fight gravity; Grade III: can fight gravity to do active joint activity, but cannot fight resistance; Grade IV: can fight a larger resistance, but weaker than normal; Grade V: normal muscle strength.

### 2.4. Clinical efficacy evaluation

Clinical efficacy was assessed using the nimodipine method as a percentage reduction in CSS score: [(pretreatment score − posttreatment score)/pretreatment score] × 100%. Basic healing: reduction rate ≥ 90%; significant improvement: 46%≤ minus rate < 90%; progress: 18% ≤ minus rate < 46%; The ratio of the sum of basic healing, obvious progress, and progress to the sample size of a single group is the total effective rate of the group.

### 2.5. Statistical analysis

SPSS26.0 software was adopted for statistical analysis of the collected data. Count data were tested by 2, and measurement data were expressed as mean ± standard deviation (*¯x* ± *s*) if they conformed to normal distribution. *t*-test for paired samples was used for intragroup comparisons, and *t*-test for 2 independent samples was used for inter-group comparisons. Data not normally distributed are expressed as median (25% quartiles, 75% quartiles) [*M*(*P*25, *P*75)], and rank data were tested by rank sum test. *P* < .05 was considered a statistically significant difference.

## 3. Research results

### 3.1. General information statistics

There were no significant differences between the 2 groups in terms of gender, age and disease duration, which were not statistically significant and comparable. The comparison of gender, age, and course of disease between the 2 groups is shown in Table 1.

**Table 1:** Comparison of gender age, and duration of disease.

Group	Gender (number of cases)		Age (years)	Duration of disease (days)
	Male	Female		
Low frequency group n = 28	21	7	56.96 ± 9.95	44.43 ± 12.57
Electroacupuncture group n = 29	23	6	60.76 ± 10.05	38.97 ± 13.74
Statistical values	*χ^2^* = 0.150		*t* = -1.433	*t* = 1.565
*P*	.698		.158	.123

### 3.2. Comparison of surface electromyographic characteristic values before and after treatment between 2 groups of patients

#### 3.2.1. Comparison of MAX and RMS scores of the first interosseous dorsal muscle before and after treatment.

Before treatment, the comparison of the values of the first interosseous dorsal muscle between the 2 groups, MAX (*Z* = −1.149, *P* = .250 > .05) and RMS (*Z* = −0.918, *P* = .359 > .05), was not statistically significant and was comparable. After treatment, the MAX and RMS values of the first interosseous dorsal muscle increased in both groups compared to the pretreatment values. The MAX and RMS of the 2 groups were statistically significant by intragroup comparison (*P* < .05); the data of the 2 groups were statistically significant by inter-group comparison for MAX (*Z* = −2.315, *P* = .021 < .0) and RMS (*Z* = −2.043, *P* = .041 < .05). The electromyoelectric characteristic values MAX and RMS of the first interosseous dorsal muscle before and after treatment of the first interosseous dorsal muscle in the 2 groups are shown in Table 2.

**Table 2:** First interosseous dorsal muscle values.

Group	First interosseous dorsal muscle			
	MAX		RMS	
	Before treatment	After treatment	Before treatment	After treatment
Low frequency group n = 28	35.80(24.29, 53.86)	97.69(47.92, 144.92)*,#	17.50(11.28, 21.21)	59.28(40.67, 83.57)*,#
Electroacupuncture group n = 29	28.17(13.47, 49.68)	47.36(33.08, 95.43)*	19.84(11.04, 29.98)	40.67(23.13, 78.53)*
*Z*	−1.149	−2.315	−0.918	−2.043
*P*	.250	.021	.359	.041

*Note*: Compared with this group before treatment,

* *P* < .05; compared with the electroacupuncture group after treatment,

#*P* < .05.

#### 3.2.2. Comparison of MAX and RMS scores before and after treatment for the short thumb adductor muscle.

Before treatment, the comparison of the thumb short abductor values between the 2 groups, MAX (*Z* = −0.040, *P* = .968 > .05) and RMS (*Z* = −0.048, *P* = .962 > .05), was not statistically significant and comparable. After treatment, the thumb short abductor MAX and RMS values increased in both groups compared to pretreatment. MAX and RMS in both groups were statistically significant by intragroup comparison (*P* < .05). The data of the 2 groups of patients were statistically significant by comparing MAX (*Z* = −6.321, *P* = .001 < .05) and RMS (*Z* = −5.611, *P* = .001 < .05) between the groups. The superficial electromyoelectric characteristic values MAX and RMS of the abductor pollicis breve before and after treatment in the 2 groups are shown in Table 3.

**Table 3:** Thumb short abductor values.

Group	Short thumb adductor			
	MAX		RMS	
	Before treatment	After treatment	Before treatment	After treatment
Low frequency group n = 28	34.45(23.53, 40.85)	100.11(91.71, 105.55)*,#	32.02(23.81, 43.78)	97.89(87.84, 107.14)*,#
Electroacupuncture group n = 29	29.42(24.95, 37.22)	78.34(66.75, 82.39)*	27.56(26.45, 39.41)	68.43(64.72, 80.26)*
*Z*	−0.040	−6.321	−0.048	−5.611
*P*	0.968	0.001	0.962	0.001

*Note*: Compared with this group before treatment,

* *P* < .05; compared with the electroacupuncture group after treatment,

#*P* < .05.

### 3.3. Comparison of CSS scores between the 2 groups of patients before and after treatment

Before treatment, the CSS scores of the 2 groups were comparable (*Z* = −1.112, *P* = .266 > .05). After treatment, the CSS scores of both groups were lower than before treatment. The data of both groups were statistically significant by intragroup comparison (*P* = .001 < .05) and statistically significant by inter-group comparison (*Z* = −2.211, *P* = .027 < .05). The comparison of CSS scores before and after treatment between the 2 groups is shown in Table 4.

**Table 4:** CSS scores.

Group	Before treatment	After treatment
Low frequency group n = 28	15.00 (13.00,16.00)	5.00 (2.25,7.00)*,#
Electroacupuncture group n = 29	15.00 (13.00,16.50)	8.00 (4.50,10.00)*
*Z*	−1.112	−2.211
*P*	.266	.027

*Note*: Compared with this group before treatment,

* *P* < .05; compared with the electroacupuncture group after treatment,

#*P* < .05.

### 3.4. Comparison of Brunnstrom motor function evaluation before and after treatment between 2 groups of patients

Before treatment, Brunnstrom motor function was comparable between the 2 groups (*Z* = −1.481, *P* = .139 > .05). After treatment, Brunnstrom motor function was higher in both groups compared with that before treatment. The data of both groups were statistically significant by intragroup comparison (*P* = .001 < .05) and statistically significant by inter-group comparison (*Z* = −2.127, *P* = .033 < .05). The comparison of Brunnstrom grades before and after treatment between the 2 groups is shown in Table 5.

**Table 5:** Brunnstrom stage.

Group	Before treatment						After treatment					
	I	II	III	IV	V	VI	I	II	III	IV	V	VI
Low frequency group n = 28	16	5	5	2	0	0	0^*#^	0^*#^	16^*#^	5^*#^	5^*#^	2^*#^
Electroacupuncture group n = 29	13	4	4	4	4	0	2^*^	13^*^	2^*^	5^*^	6^*^	1^*^
*Z*	−1.481						−2.127					
*P*	.139						.033					

*Not*e: Compared with this group before treatment,

*P < .05; compared with the electroacupuncture group after treatment,

#*P* < .05.

### 3.5. Comparison of MAS grading before and after treatment between 2 groups of patients

Before treatment, the MAS classifications of the 2 groups were comparable (*P* = .946 > .05, *Z* = −0.068). After treatment, the MAS classifications of both groups were lower than before treatment. The data of the 2 groups were statistically significant by intragroup comparison (*P* < .05) and statistically significant by inter-group comparison (*P* = .001 < .05, *Z* = −4.344). The comparison of MAS grades before and after treatment between the 2 groups is shown in Table 6.

**Table 6:** MAS grade.

Group	Before treatment					After treatment				
	Grade 0	Grade 1	Grade 2	Grade 3	Grade 4	Grade 0	Grade 1	Grade 2	Grade 3	Grade 4
Low frequency group n = 28	0	6	22	0	0	21*#	7*#	0*#	0*#	0*#
Electroacupuncture group n = 29	0	6	23	0	0	6*	15*	8*	0*	0*
*Z*	−0.068					−4.344				
*P*	.946					.001				

*Note*: Compared with this group before treatment,

* *P* < .05; compared with the electroacupuncture group after treatment,

#*P* < .05.

### 3.6. Comparison of Lindmark hand function scores between the 2 groups of patients before and after treatment

Before treatment, Lindmark hand function scores were comparable between the 2 groups (*P* = .693 > .05, *Z* = −0.394). After treatment, Lindmark hand function scores were higher in both groups compared to before treatment. The data of the 2 groups were statistically significant by intragroup comparison (*P* < .05) and statistically significant by inter-group comparison (*P* = .007 < .05, *Z* = −2.674). The comparison of Lindmark hand function scores before and after treatment between the 2 groups is shown in Table 7.

**Table 7:** Lindmark hand function scores.

Group	Before treatment	After treatment
Low frequency group n = 28	11.00 (9.25,13.00)	21.00 (20.00,22.75)*,#
Electroacupuncture group n = 29	12.00 (9.00,13.50)	20.00 (18.00,21.50)*
*Z*	−0.394	−2.674
*P*	.693	.007

*Note*: Compared with this group before treatment,

* *P *< .05; compared with the electroacupuncture group after treatment,

#*P* < .05.

### 3.7. Comparison of Lovett muscle strength classification before and after treatment between the 2 groups

Before treatment, the Lovett muscle strength classifications of the 2 groups were comparable (*P* = .316 > .05, *Z* = 1.003). After treatment, the Lovett muscle strength classifications of both groups were higher than before treatment. The data of the 2 groups were statistically significant by intragroup comparison (*P* < .05) and statistically significant by inter-group comparison (*P* = .031 < 0.05, *Z* = −2.156). The comparison of Lovett muscle strength classification before and after treatment in the 2 groups is shown in Table 8.

**Table 8:** Lovett muscle strength classification.

Group	Before treatment						After treatment					
	0	I	II	III	IV	V	0	I	II	III	IV	V
Low frequency group n = 28	0	0	7	11	10	0	0*#	0*#	0*#	0*#	13*#	15*#
Electroacupuncture group n = 29	0	0	5	10	14	0	0*	0*	0*	7*	12*	10*
*Z*	−1.003						−2.156					
*P*	.316						.031					

*Note*: Compared with this group before treatment,

* *P* < .05; compared with the electroacupuncture group after treatment,

#*P* < .05.

### 3.8. Comparison of clinical efficacy between 2 groups of patients

After treatment, the efficiency rates of the low-frequency and EA groups were 93.67% and 82.61%, respectively, with statistically significant differences (*P* = .035 < .05). The comparison of clinical efficacy before and after treatment between the 2 groups is shown in Table 9.

**Table 9:** Clinical efficacy.

Group	Cured	Significant improvement	improvement	Ineffective	Total efficiency	*Z*	*P*
Low frequency group n = 28	7	17	2	2	92.86%*	.035	−2.114
Electroacupuncture group n = 29	4	13	6	6	79.31%		

*Note*: Compared with the electroacupuncture group,

* *P* < .05.

## 4. Discussion

Thumb-to-finger dysfunction is a difficult point in the treatment of stroke sequelae, affecting upper limb function and grasping ability. In this study, thumb-to-finger dysfunction was mainly due to decreased activation of the extensor and flexor muscles of the fingers^[18,19]^ and increased flexor tone,^[20]^ rather than motor planning problems. Cinzia Calautti et al^[21]^ believe that not only does finger tapping speed decrease after stroke, but also the regularity of percussion is impaired, resulting in reduced flexibility. Motor neurons cannot be activated and regulated after a stroke to innervate all muscles, not just the extensor fingers. The relationship between the ability to stretch fingers and the ability to grasp may not be strictly linear, and other muscles used for grasping may not be sufficiently activated or only selectively activated. Helleana Eschmann et al^[22]^ compared the amount of thumb and finger movement of healthy people and stroke patients for a long time, and found that the finger movement free time of patients with hand dysfunction after stroke was greater than that of healthy people, and the rhythm of thumb and finger movement was lower than that of healthy people. Kathryn S Hayward et al^[23]^ believe that loss of arm and hand function after stroke is the main cause of disability, and patients are eager to recover, but the goal of permanent restoration of arm and hand function has not been achieved, and high-dose, behavior-centered rehabilitation interventions are still needed. Therefore, improving the movement, rhythm and precision of the patient’s hands and fingers is the key to treatment. The previous research of our group^[8]^ showed that low-frequency acupoint electrical stimulation (LFES) therapy can better adapt to the abnormal movement pattern of hemiplegic patients, and through the alternating stimulation method of flexor and extensor muscles, that is, the flexor and extensor muscle stimulation ratio is 1:2, it can enhance the cooperation between the active and passive muscles on the paralyzed side of stroke patients and promote the recovery of thumb-to-finger movements. Marc P Powell et al^[24]^ reported a human trial using electrical stimulation to activate cervical spinal cord circuits to improve arm and hand movements in patients with upper limb paralysis after stroke. Two patients had 2 electrodes implanted over 29 days, located in the dorsolateral space of the spinal cord, with the aim of enhancing motor neurons that control the arms and hands. The results showed that electrical stimulation could improve strength, speed, and functional movement in patients, allowing them to perform tasks that would otherwise be impossible. Two patients maintained some improvement even after stopping stimulation and there were no serious adverse events. This study provides preliminary evidence for the treatment of upper limb paralysis after stroke, suggesting that spinal cord stimulation may be a beneficial method of rehabilitation. This project aims to observe the clinical efficacy of LFES in improving thumb-to-finger action after stroke, and to provide new diagnostic and therapeutic ideas for the clinical treatment of thumb-to-finger dysfunction. In this study, it was believed that the yin and yang imbalance of qi and blood was the pathogenesis of this disease,^[25]^ and the acupoints of the hand yang ming meridian, the hand yin pericardial meridian and the hand taiyin lung meridian were selected to balance yin and yang and harmonize qi and blood. Shouyang Ming Jing treats upper limb failure,^[26]^ and selects Shousanli and Hegu acupoints; Waiguan acupoint through yang vein, adjust the 6 yang meridian qi^[27]^; Neiguan acupoint through the yin vein, regulating the yin meridian qi and blood^[27]^; Haomen acupoint can regulate qi and blood^[28]^; Stimulating Hegu acupoint can make Yangming through qi^[29]^; Yuji points regulate the motor function of the thumb.^[30]^ From an anatomical point of view, the selected acupoint corresponds to the muscles and motor function of the hand. The Shousanli and Waiguan points of the hand are the first group of low-frequency electrical stimulation in the extensor muscle group, so that the wrist and back are extended and the thumb is extended; Neiguan and Ximen acupoints are the second group of low-frequency electrical stimulation, which is mainly responsible for 2 to 4 finger flexion and forearm pronation and wrist flexion; Hegu and Yuji Cave are the third group of low-frequency electrical stimulation, which can make the thumb index finger to finger to complete the precise pinching action. The first interosseous dorsal muscle and abductor pollicis brevis are the key muscles for precise pinching.

The results showed that the 2 therapies improved the pinching function between the thumb and 2 fingers, the flexion exercise of the interphalangeal joint extension exercise of the thumb, the neurological function defect after stroke, the grading of hand motor function, the degree of upper limb spasm, the muscle strength of the hand, muscle tone, the coordination function between each other and the muscle strength classification of patients after stroke, and the efficacy of the low-frequency group was significantly better than that of the EA group (*P* < .05). The treatment plan suggesting that the treatment plan of LFES has a better degree of improvement than that of EA in patients with hand dysfunction after stroke.

This study explores the clinical effect of LFES therapy in the treatment of this disease, but there are some shortcomings and prospects. The main problems are: failure to compare and follow up the efficacy of cycles, and the inability to determine the optimal treatment cycle and long-term efficacy; The mechanism was not explored, only clinical efficacy was proved, and the mechanism of action and evaluation of safety and scope of adaptation could not be explained; Trials were conducted only in a single center, with small sample sizes, possible selection bias and regional variation, affecting our confidence in the results. It is recommended to improve the following in the future: regular follow-up of patients to clarify long-term clinical efficacy; Increase the comparison of clinical efficacy of cycles and clarify the optimal treatment cycle; Conduct animal experiments to prove scientific and normative nature, and conduct mechanism discussions; Adopt multi-center, large-sample clinical trials to improve rigor and objectivity; Studies were conducted in different regions and populations to analyze applicability and effectiveness, and to explore differentiation and individualization.

## 5. Conclusion

Both FLES and EA showed efficacy in the reconstruction of motor function of the thumb-index finger movements, as evidenced by the increase in MAX and RMS values of the first interosseous dorsal muscle and thumb short extensor muscle, the decrease in CSS score, the increase in Brunnstrom motor function score, the decrease in MAS classification, the increase in Lindmark hand function score, and the increase in Lovett muscle strength classification, and the improvement of FLES therapy was more obvious and the efficacy was better than that of EA therapy, which is worthy of clinical promotion.

## Author contributions

**Conceptualization:** Dongyan Wang.

**Data curation:** Xu Dong, Hong Huo, Ying Zhang, Jing Song.

**Writing – original draft:** Xue Xia.

**Writing – review & editing:** Xue Xia, Dongyan Wang.
